# Validation of an LCMS method for stability evaluations of piperine in *Murchita utpalashatpalaghrita*

**DOI:** 10.1016/j.jaim.2025.101286

**Published:** 2026-02-04

**Authors:** Ashutosh Gupta, Moumita Saha, Abhishek Ravindra Malandkar, Aditya Dev Rajora, Shivani Kunkalienkar, J. Dinesh Nayak, Sudheer Moorkoth

**Affiliations:** a Department of Pharmaceutical Quality Assurance, Manipal College of Pharmaceutical Sciences, Manipal Academy of Higher Education, Manipal, 576104, Karnataka, India; b Department of Post Graduate Studies in Rasa-Shastra & Bhaishajya Kalpana, Muniyal Institute of Ayurveda Medical Sciences, Manipal, 576104, Karnataka, India

**Keywords:** Piperine, LCMS, Murchita, Stability, *Utpalashatpala ghrita*

## Abstract

**Background:**

*Utpalashatphala ghrita* (USG) is a traditional ayurvedic medicine containing piperine as its main chemical constituent responsible for the activity. *Murchita* is an ayurvedic process to detoxify and purify the lipids (ghee, oil). It involves heating ghee with specific ayurvedic ingredients for a specified duration and temperature. It is essential to understand the stability of piperine during and after the murchita process.

**Objective:**

The objective of this study was to develop and validate an LCMS method to assess the stability of piperine after the *murchita* process.

**Method:**

LCMS system with electrospray ionization and ion-trap as the analyser was used to estimate the piperine content. Analysis was carried out in the multiple reaction monitoring (MRM) mode. Chromatographic mobile phase composed of a mixture of 55 % formic acid (0.1 %) in Milli-Q water (pH 3) and 45 % methanol. The LC-MS method was validated as per ICH Q2 (R2) guidelines. Stability of *ghrita* was assessed at various stability conditions.

**Result:**

The developed LCMS method was accurate, precise and linear over a range of 0.5–32 μg/mL. LOQ of the method was 125 ng/mL and the run time was 10 min. *Utpalashatphala* ghrita (USG) and *Murchita- Utpalashatphala ghrita* (M-USG) were prepared as specified under *Bhaishajya Ratnavali*. Our study revealed that the murchita process degraded piperine by 26 %. However, the stability of piperine was found to be better in the murchita ghrita (M -USG), compared to the unprocessed USG.

**Conclusion:**

Study effectively demonstrated that the *murchita* process enhanced the long-term stability of piperine.

## 1 Introduction

This Indian traditional medical system places equal importance on the use of herbal remedies in addition to the idea of self-healing [1]. The World Health Organization (WHO) indicates that 70–80 % of the world's population gets their medical treatment from nonconventional sources, mostly herbal sources [2]. The major reasons for the public's interest in complementary and alternative systems of medicine are the absence of effective treatments for many chronic conditions, high cost of new medications, increasing microbial resistance and increased adverse effects of synthetic therapeutics [3].

Ayurveda is based on three main pillars such as *Hetu* (causative factors), *Linga* (symptoms), and *aushadha* (medicines). Among this *aushadha* plays important role in curing diseases as well as maintaining good health. Important dosage forms for administering *aushadha* as per Ayurveda include *churna* (fine powder), *vati* (tablets), *ghrita* (ghee or clarified butter), *taila* (oil), *arka* (liquid herbal distillate), *asava* (fermented liquid medicine), *arishta* (fermented herbal medicine), *lavana* (salt), kshar (water soluble ashes of medicinal plant), *avaleha* (semisolid herbal preparetion), *malahara* (ointment for wound dressing), and *gugulu* (oleo resin). Among these *ghrita* (ghee containing preparation) is a most widely used medicated dosage form. *Ghrita* is one of the best ayurvedic formulation, which is easily assimilated in the body. Before the preparation of medicated ghee (*aushadha* *siddha ghrita*), *ghrita murchita samskara* (processing of ghee) is mentioned in *Bhaishajya Ratnavali* to reduce amadosha (moisture content), *durgandhata* (bad odour) and to enhance *viryata* (potency) of the *sneha* (fat). *Murchita* process imparts good colour, odour, and minimizes rancidity. It also facilitates better dissolution of bio constituents in *ghrita* [4]. The fact that the *murchita* process is a time-consuming step makes the ayurvedic pharmaceutical companies to skip this *murchita* step to avoid delays and reduce the production cost.

*Utpalashatphala ghrita* (USG) is a traditional ayurvedic medicine used for conditions such as spleen disorder, cough, fever and dyspnea. Major ingredients of USG are volatile oils, essential oils, and piperine (PIP). PIP is a bioactive alkaloid found in black pepper (*Piper nigrum*) and long pepper (*Piper longum*). It is widely recognized for its therapeutic benefits in traditional and modern medicine. One of its most significant property is its ability to enhance bioavailability by inhibiting drug-metabolizing enzymes (CYP450) and P-glycoprotein [5]. PIP also exhibits anti-inflammatory, antioxidant, and immunomodulatory effects [6], making it beneficial in chronic diseases such as arthritis, neurodegenerative disorders, and cardiovascular conditions. Its antimicrobial and gastroprotective properties also contribute to digestive health by stimulating digestive enzymes and improving gut function [7]. Studies have also highlighted its anticancer potential, which induces apoptosis and tumour growth inhibition [8]. Furthermore, PIP plays a role in weight management and metabolic health by enhancing thermogenesis and lipid metabolism, which may aid in fat reduction [9]. Due to its diverse pharmacological actions, PIP is widely used as an adjuvant in herbal and pharmaceutical formulations to enhance therapeutic efficacy.

The stability of PIP is a major factor affecting the shelf life of utpalashatpala ghrita. In this study, we prepared *utpalashatpala ghrita* (USG) and murchitha *utpalshatpala ghrita* (M-USG) and then evaluated the effect of the *murchita* process on the stability of PIP. As of this date, there are no analytical methods available to evaluate the stability of PIP from ayurvedic ghee preparations. A validated LCMS method was developed to quantify PIP from ghee preparations. Evaluating PIP concentration ensure consistency between batches, verifies the presence of authentic ingredients, and supports pharmacological claims. Since the lipophilic nature of PIP allows it to integrate well with *ghrita*, a traditional lipid-based medium used in ayurveda for drug delivery, quantifying PIP content not only validates the potency and stability of the preparation but also aligns traditional knowledge with modern analytical standards, bridging the gap between ancient practice and contemporary pharmaceutical quality assurance.

## 2 Materials and methodology

### 2.1 Materials

Piperine (light yellow powder, purity >97 %) sourced from Sigma-Aldrich Chemicals Private Limited, Bangalore India. Himedia Labs Pvt. Ltd., (Mumbai, India) supplied the hydrogen peroxide (30 %) and the sodium hydroxide pellets (98 % purity). Orthophosphoric acid (88 %) was purchased from Merck Ltd. Mumbai, India. LCMS grade methanol (MeOH) and acetonitrile (ACN) were purchased from Finar Ltd (Ahmedabad, India). High pure water (18.2 MΩ cm resistivity, Milli-Q) required for the LCMS analysis was produced in-house using Direct-Q ® 3 water purification system, Merck-Millipore Corporation, Billerica, USA. Riviera Glass Pvt. Ltd. (Mumbai, India) provided 0.22 μm membrane filters. Formic acid and ammonium formate was purchased from Merck Labs Pvt. Ltd. in Mumbai, India. The Kinetex C18 column (100 mm × 3 mm × 2.6 μm, 100 Å) was purchased from Phenomenex (Hyderabad, India). The LCMS grade chemicals were employed in the method development and validation processes. Raw herbal drugs for the preparation of formulation were collected from known sources, identified by experts of Department of Dravyaguna, Muniyal Institute of Ayurveda Medical Sciences, Manipal, and analyzed in Department of Research and Development, Muniyal Institute of Ayurveda Medical Sciences, Manipal. Cow ghee was purchased from the local vendor. In order to avoid variability in the formulations that could arise from ghee composition from multiple sources, we restricted our procurement of ghee from one vendor only.

### 2.2 Instrumentation

A Thermo Scientific (Massachusetts, US) LC-MS with Dionex Ultimate 3000 liquid chromatograph hyphenated with an LTQ XL linear ion trap mass spectrometer was used. A heated electron spray ionization (HESI) was used as the ion source. MS/MS and chromatographic method development was performed using Xcaliber (Massachusetts, US) and Chromeleon (Massachusetts, US) software, respectively.

### 2.3 Analytical method development and validation for quantification of piperine

#### 2.3.1 Preparation of mobile phase and standard solutions

Mobile phase (MP): The MP is composed of a mixture of 55 % formic acid (0.1 %) in Milli-Q water (pH 3) and 45 % methanol. The buffer was then filtered through a 0.22 μm membrane filter before sonicating it.

Stock and standard solutions: A stock solution of 1 mg/mL was prepared by dissolving 10 mg of PIP into 5 mL of methanol using bath sonication. The volume was then brought up to 10 mL using methanol to make the stock solution. By diluting 1 mL of the stock solution to 10 mL in another volumetric flask, a working stock solution of 100 μg/mL of PIP was prepared. Eight calibration solutions in the range of 0.5–32 μg/mL were then prepared from the working stock solution by serial dilution. QC solutions of PIP (HQC, MQC and LQC) were also prepared from the working stock.

#### 2.3.2 LCMS method development and validation

Variables that affect separation in LCMS such as stationary phase, mobile phase ratio, mobile phase pH, mobile phase flow rate, temperature of column oven, ionization, spray voltage and injection volume, were optimized by performing LCMS trial runs. The optimized LCMS method conditions are presented in the results section. The developed method was then validated for its sensitivity, specificity, linearity, Limit of Detection (LOD), Limit of Quantification (LOQ), accuracy, precision and robustness as per the ICH Q2 (R2) guidelines [[10], [11], [12]].

### 2.4 Preparation of the ayurvedic formulations M-USG and USG

#### 2.4.1 Procedure for the preparation of murchita ghrita

*Ghrita murchita* was carried out as per the literature reference given in Bhaishajya Ratnavali, jwarachikitsa verse no.1285. Ingredients used in for the preparation of *murchita ghrita* is presented in Table 1.

**Table 1:** Ingredients present in the *ghrita murchita*.

Sl No.	Ingredients	Part used	Botanical name	Weight
1.	*Haritaki*	Dried fruit	*Terminalia Chebula*	68.79 g
2.	*Vibhitaki*	Dried fruit	*Terminalia belerica*	68.79 g
3.	*Amalaki*	Dried fruit	*Emblica officinalis*	68.79 g
4.	*Haridra*	Rhizome	*Curcuma longa*	68.79 g
5.	*Musta*	Root tuber	*Cyperus rotundus*	68.79 g
6.	*Matulunga*	Fruit rind	*Citrus medica*	68.79 g
7.	*Jala*	–	*Water*	4.400 L
8.	*Ghrita*	–	Cow ghee	1100 g

One *prastha* (unit of measurement, weight equivalent to 640 g) of *Goghritham* (cow-ghee) is heated at Madhyama (moderate or middle state) agni for 10 min, and allowed to cool down on its own. Then *kalka* (herbal paste) made of *haritaki*, *amalaki*, *vibhitaki*, *haridra*, *musta* taken each in one *pala* (unit of measurement, weight equivalent to 40 g) quantity all mixed together and made into kalka form by matulunga swarasa (extracted juice). This *kalka* & *jala* (water) is then added with *ghrita* slowly and heated on *mandagni* (slow fire) till bubbles and waves disappear. Sometimes while adding *kalka*, *ghrita* may come out of the pot due to some kind of reaction. For this *kalka* should be added after cooling the *ghrita* and then again it should be heated for some time and filtered. It is claimed to remove *amadosha* (moisture content) and induce some potency in the *ghrita*. This prepared *Murchita* *ghrita* is used as base for preparation of M-USG [13,14].

#### 2.4.2 Procedure for the preparation of *Murchita Utpalashatphala ghrita* (M-USG)

M-USG was prepared by the following procedure: *Pippali, Pippalimula, Chavya, Chitraka, Shunthi* and *Yavakshara* were taken and made fine powder and then converted into *kalka* form. *Murchita ghrita*, which was prepared previously, was taken in *sneha patra* (vessel or container for oil/ghee) and melted. To this melted *ghrita*, *kalka*, *go ksheera* (cow-milk), and *jala* were added & heated on *madhyama agni* on gas stove to evaporate the aqueous part. The contents were stirred regularly to avoid burning. Heating is continued till the *ghrita sidhi lakshana* appeared (*ghrita siddhi lakshana*s – signs of completion of *ghrita* *paka*). At this stage *ghrita* was filtered through cloth and preserved [14]. Ingredients used for the preparation of *Murchita Utpalashatphala ghrita* are shown in Table 2.

**Table 2:** Ingredients present in the *Murchita Utpalashatphala ghrita*.

Sl No.	Ingredients	Botanical name	Weight
1.	*Pippali*	*Piper longum* (fruit)	21.875 g
2.	*Pippali moola*	*Piper longum* (roots)	21.875 g
3.	*Chavya*	*Piper Chaba Hunter.*	21.875 g
4.	*Chitraka*	*Plumbago zeylanica*	21.875 g
5.	*Shunthi*	*Zingiber officinale* Rose.	21.875 g
6.	*Yavakshar*	Alkali of Hordeum vulgare	21.875 g
7.	*Godugdha*	Cow milk	350 mL
8.	*Jala*	Water	1050 mL
9.	*Ghrita* (*Murchita* ghee)	–	350 mL

#### 2.4.3 Procedure for the preparation of *utpalashatphala ghrita* (USG)

USG was prepared in the same way as M-USG. Here in this procedure instead of *Murchita ghrita*, only plain cow ghee is used. All other ingredients and methods of preparation is same like M-USG (Table 3).

**Table 3:** Ingredients present in the *Utpalashatphala ghrita*.

Sl No.	Ingredients	Botanical name	Weight
1.	*Pippali*	Piper longum (fruit)	21.875 g
2.	*Pippali moola*	Piper longum (roots)	21.875 g
3.	*Chavya*	Piper Chaba Hunter.	21.875 g
4.	*Chitraka*	Plumbago zeylanica	21.875 g
5.	*Shunthi*	Zingiber officinale Rose.	21.875 g
6.	*Yavakshar*	Alkali of Hordeum vulgare	21.875 g
7.	*Godugdha*	Cow milk	350 mL
8.	*Jala*	Water	1050 mL
9.	*Ghrita*	Cow ghee	350

### 2.5 Procedure for extraction of piperine from the *ghrita*

For the extraction of PIP from the prepared ghrita formulations, 0.1 g ghrita was accurately weighed and vortexed with 1 mL of chloroform for 2 min to facilitate the dissolution of PIP, which is a lipophilic compound. This step ensures efficient extraction of PIP from the lipid-based matrix of the *ghrita*. The mixture was then centrifuged at 10,000 rpm for 15 min to separate the solid and insoluble lipid components from the chloroform layer containing the dissolved PIP. From the supernatant, 100 μL was carefully collected and further diluted to 1 mL of mobile phase to make it suitable for LC-MS analysis. The final sample was injected into the LC-MS system for the quantification of PIP [5].

### 2.6 Evaluation of stability of the prepared *ghrita*

The evaluation of PIP content in the ghrita formulation was used as a stability marker to evaluate the chemical integrity of the product over time. Quantification of PIP was done using the validated LCMS method, ensuring accuracy and reproducibility of the results. The stability evaluation of USG and M-USG was performed at four conditions, such as room temperature, refrigerator temperature (2–8 °C), intermediate stability testing conditions (30 ± 2 °C/65 ± 5 % RH), and at the accelerated stability testing conditions (40 ± 2 °C/75 ± 5 %) respectively for 6 months following the ICH Q1A (R2) stability testing guideline. The samples were collected and analyzed at 0, 1, 2, 3, and 6 months in triplicates to check the degradation profile of PIP under each conditions [15]. This systemic evaluation helped in understanding the influence of environmental factors on the stability of PIP and, by extension, on the overall quality and shelf life of the ghrita formulation. The stability study was performed in three replicates at each time point to obtain necessary statistical power for the study.

### 2.7 Greenness of developed method

The greenness of the analytical method was performed using the AGREE software (Analytical Greenness Metric Approach and Software) [16,17]. This technique takes into account 12 factors, each of which is given a score between 0 and 1, with higher numbers denoting a greener approach.

## 3 Results and discussion

**Fig. 1:**
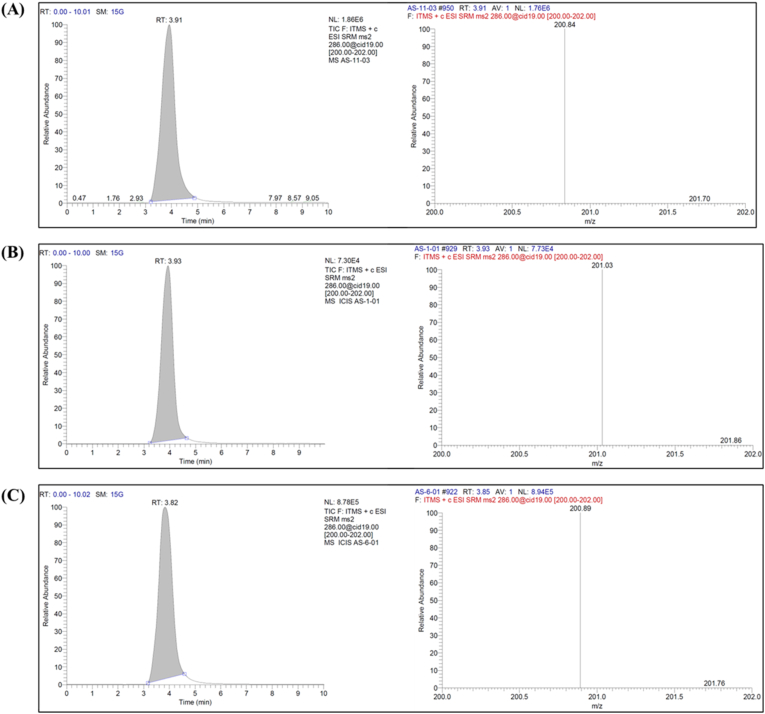
Chromatogram and spectrogram of (A) pure piperine, (B) piperine extracted from Utpalashatphala ghrita (USG), (C) piperine from *Murchita Utpalashatphala* (M-USG).

Piperine is an alkaloid with an amide bond, conjugated double bonds, and a methylenedioxyphenyl ring having a logP value of 3.0. Based on the logP value a nonpolar stationary phase, Kinetex C18 column (100 mm × 3 mm × 2.6 μm, 100 Å) was chosen. PIP is moderately nonpolar and soluble in organic solvents like methanol and acetonitrile. It has a pKa value of 12.3 because of the weakly basic amide nitrogen in the piperine molecule and behaves like a neutral (non-ionized) molecule at physiological pHs (3–8 pH). Accordingly, a mobile phase composition consisting of 0.1 % formic acid in Milli-Q water (pH 3.0) and methanol were selected as the aqueous phase and organic phase, respectively. A 55:45 ratio of aqueous to organic phase resulted in a good separation. The chromatogram obtained at the optimized conditions are shown in Fig. 1. Piperine showed the response at a retention time of 3.9 min. Fig. 1A is the chromatogram obtained from pure piperine, while Fig. 1B is that of piperine extracted from USG and Fig. 1C is of piperine extracted from M-USG. The mass spectrometric response that can be seen in the chromatogram is in the range of E4 to E6, proving that the method is highly suitable for quantification of piperine from USG and M-USG. Employment of Selective Reaction Monitoring (SRM) analysis allowed us to selectively analyze the fragment at *m/z* 201, which is obtained with a collision of PIP *m/z* 286 with a collision energy of 19 eV. This provided highly selective analysis of PIP from other components present in the complex ayurvedic formulation.

### 3.1 Optimized chromatography conditions

Mobile phase composed of a mixture of 0.1 % formic acid (pH 3.0) with milli-q water and methanol (55:45). The flow rate was set at 0.2 mL/min. The column and autosampler temperature were maintained at 25 °C and 4 °C, respectively. The injection volume was 4 μL and the total run time was 10 min. The Kinetex C18 column (100 mm × 3 mm × 2.6 μm, 100 Å) from Phenomenex, was used for the analysis.

### 3.2 Optimized mass spectrometry conditions

The ionization source of the mass spectrometer was a heated electrospray ionization (ESI), that was run under the following conditions: spray voltage (4.00 kV), heater temperature (350 °C), capillary voltage (30 V), capillary temperature (250 °C), tube lens offset voltage (55 V), sheath gas flow rate (30 arb), auxiliary gas flow rate (10 arb), and sweep gas flow rate (0 arb) units. The mass spectrometer was operated in positive/negative switching mode in the full scan mode. The mass spectrometer was operated in positive/negative switching mode with selected reaction monitoring (SRM). The SRM transitions and the respective collision energy (CE) used for piperine were as follows: PIP (*m/z* 286.00 → 201.3, MOI: (+) CE: 19 eV).

#### 3.2.1 Validation of developed LCMS method for piperine

The developed LCMS method was validated as per ICH guidelines [10]. The results of validation are provided in Table 4. The selectivity evaluation helps to check the interference with any other ingredients in the formulation, excipient, matrix and solvent with the peak of PIP. The MS chromatogram of pure PIP, USG, M-USG illustrated in Fig. 1, shows no interferences proving the selectivity of the method for analysis of piperine.

**Table 4:** Validation data of the analytical method of piperine.

**Parameters**	**Validation results**		
Range (μg/mL)	0.5–32		
Regression equation	y = 39486x – 4236.7		
Linearity Correlation coefficient (r^2^)	0.9999		
LOD (ng/mL)	90		
LOQ (ng/mL)	125		
**Accuracy**	**% Recovery**		
LQC	99.78		
MQC	101.81		
HQC	101.34		
**Precision**	**Repeatability**	**Intra-day**	**Inter day**
%RSD	0.13	0.64	1.6

**Note:** LQC: Lower Quality control, MQC: Mediam Quality control, HQC: High Quality control, LOD: Limit of Detection, LOQ: Limit of Quantification.

**Fig. 2:**
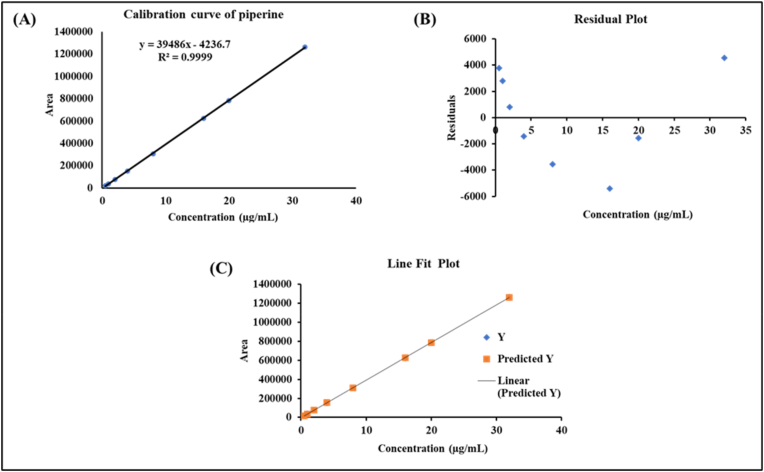
Curve plotted between peak area vs piperine concentration (A) calibration curve, (B) residual plot, and (C) line fit plot.

The method showed an accuracy of 99.78 % (LQC), 101.81 % (MQC) and 101.34 % (HQC), proving that method is accurate at all QC levels. Accuracy was well within the acceptance limits of accuracy as set by ICH of ±15 % for ayurvedic formulations. The precision of the method as represented by the % RSD showed 0.13 % (repeatability), 0.64 % (intraday precision), and 1.6 % (inter day) proving the very high precision of the method and proves the ruggedness of the method. The limit of quantification of the method was found to be 125 μg/mL proving the sensitivity of the method for the analysis. This high sensitivity of the method clearly indicate that the method has the capability of quantifying PIP that are normally present in any of the ayurvedic formulation. The method also showed good linearity (R^2^ = 0.999) over the analytical range of 0.50–32 μg/mL. The calibration curve, residual plot and line fit plot has been depicted in Fig. 2A, B, and 2C. The robustness for area and retention time checked at all QC levels by slight changes in pH, mobile phase composition and temperature of the column oven showed an accuracy and precision within the acceptance limits proving the robustness of the method.

#### 3.2.2 Stability evaluation of piperine in *utpalashatphala ghrita* and *utpalashatphala* ghrita fortified by *murchita*

Initially, the content of PIP in USG and M-USG was determined using the validated LCMS method before subjecting them for stability evaluation (zero timepoint). The PIP concentration in the ghrita was found to be 121.46 ± 1.72 μg in 0.1 gm of USG and 86.42 ± 0.52 μg in 0.1 gm of M-USG respectively. The stability evaluation of USG and M-USG were performed at four conditions: room temperature, refrigerator temperature, intermediate stability testing conditions (30 ± 2 °C/65 ± 5 % RH), and at accelerated stability testing conditions (40 ± 2 °C/75 ± 5 % RH) respectively for 6 months as per ICH Q1A (R2) guidelines. Content of piperine in USG and M-USG were determined at 1, 2, 3 and 6 months timepoints using the validated method. The results of stability testing are provided in Table 5.

**Table 5:** Piperine content in the ghrita (μg in 0.1 gm of ghrita) at various timepoints and stability study conditions.

Time points in months	Piperine content (μg/0.1 gm of ghrita±SD) (n = 3)							
	Room temperature condition (25 ± 5 °C)		Intermediate conditions (30 ± 2 °C/65 ± 5 % RH)		Accelerated conditions (40 ± 2 °C/75 ± 5 % RH)		Refrigerator condition (2–8 °C)	
	USG	M-USG	USG	M-USG	USG	M-USG	USG	M-USG
**0**	121.46 ± 1.73	86.42 ± 1.99	121.46 ± 1.73	86.42 ± 1.99	121.46 ± 1.73	86.42 ± 1.99	121.46 ± 1.73	86.42 ± 1.99
**1**	118.86 ± 1.12	84.97 ± 0.57	115.90 ± 1.59	84.26 ± 0.78	100.11 ± 3.02	83.40 ± 2.10	120.64 ± 0.15	86.27 ± 0.03
**2**	110.48 ± 1.99	83.76 ± 0.89	107.38 ± 3.02	81.07 ± 1.50	86.73 ± 4.67	76.74 ± 2.80	118.98 ± 0.75	85.49 ± 0.18
**3**	99.08 ± 3.61	82.92 ± 1.15	96.86 ± 3.99	76.99 ± 1.84	72.71 ± 3.41	71.80 ± 3.64	117.30 ± 1.26	84.46 ± 0.37
**6**	89.29 ± 3.29	81.03 ± 2.49	79.58 ± 3.65	73.25 ± 2.51	49.21 ± 4.70	63.75 ± 3.26	113.58 ± 2.40	83.88 ± 0.56

**Note:** Analysis was conducted in triplicate (n = 3).

In our study we observed that the initial PIP content in M-USG was lower than that found in USG. This difference could be attributed to the loss of PIP during the murchita process, which involves additional heating and processing steps that can lead to PIP degradation. This observation was corroborated by the reports of *Nisha* et al., and *Cho* et al., where they have shown that the prolonged exposure to higher temperatures shortens its degradation half-life, which follows first order kinetics resulting in faster degradation of PIP [18,19]. However, the stability studies conducted under various storage conditions showed a lower degradation of PIP in M-USG compared to USG, indicating that M-USG possesses significantly improved chemical stability in the final formulation, preserving the PIP content in the formulation.

##### 3.2.2.1 Stability at room temperature

**Fig. 3:**
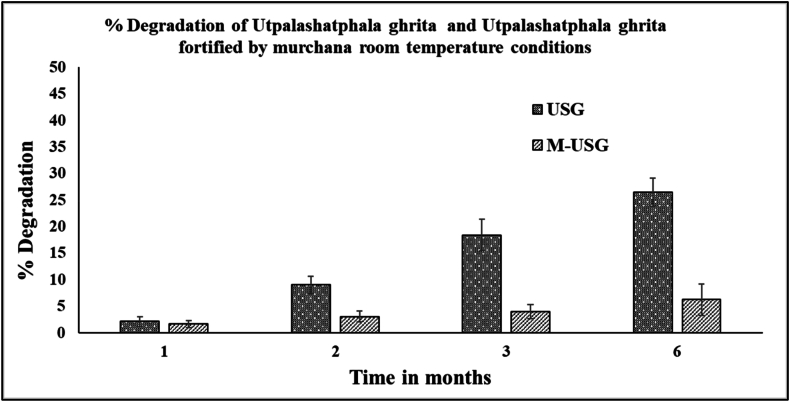
Pictorial representation of results stability study of piperine in USG and M-USG at room temperature conditions.

The stability study performed at room temperature revealed that the concentration of PIP decreased in USG in comparison to M-USG. After one month, PIP degraded by 2.14 % in USG compared to degradation of 1.67 % in M-USG. After two months the concentration of PIP was reduced by 9.04 % in USG and 3.07 % in M-USG. In the third month, the degradation of PIP increased to 18.42 % in USG while it was 4.04 % in M-USG. After 6 month the concentration of PIP had decreased drastically by 26.48 % in USG, while in M-USG, the degradation was only 6.23 %. Fig. 3 provides a visual representation of the % of degradation of PIP in USG and M-USG. The results show that M-USG retains PIP significantly better than USG during storage at room temperature, with much lower degradation rates observed over six months. This indicates that the *murchita* process improves the chemical stability of PIP in ghrita formulations. Consequently, M-USG can offer a longer shelf-life and better preservation of PIP compared to USG.

##### 3.2.2.2 Stability at intermediate stability conditions (30 ± 2 °C/65 ± 5 % RH)

**Fig. 4:**
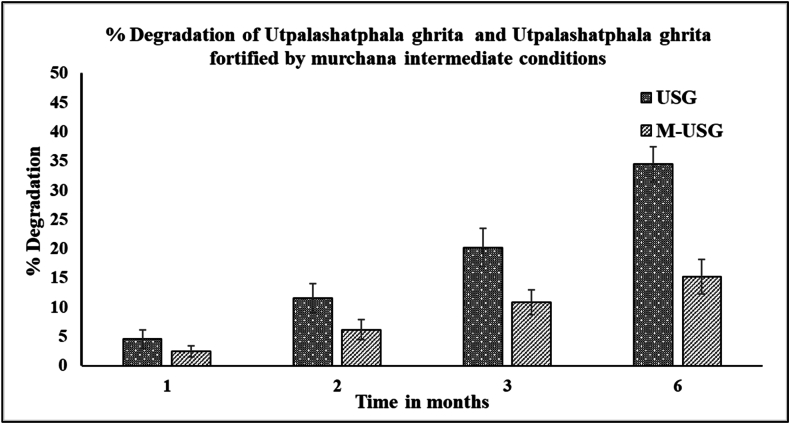
Pictorial representation of stability study of piperine in USG and M-USG at Intermediate conditions.

According to the stability study conducted at intermediate stability conditions, the concentration of PIP was lower in USG than in M-USG. After one month, the concentration of PIP was degraded by 4.56 % in USG, whereas it was degraded by 2.48 % in M-USG. After two months, the concentration of PIP was reduced by 11.58 % in USG and 6.18 % in M-USG. After three months, the degradation of PIP increased to 20.24 % in USG while it was only 10.90 % in M-USG. The concentration of PIP had decreased by 34.48 % in USG, while it was only 15.23 % in M-USG after six months, as shown in Fig. 4. Under intermediate conditions, degradation was higher compared to room temperature conditions, for USG and M-USG. However, the PIP loss was higher in USG. This could be explained due to the higher temperature and humidity of the intermediate study conditions. Over a period of six months, PIP loss in USG was more than double that in M-USG, highlighting the enhanced protective effect of the murchita process on active ingredient preservation.

##### 3.2.2.3 Accelerated condition (40 ± 2 °C/75 ± 5 % RH)

**Fig. 5:**
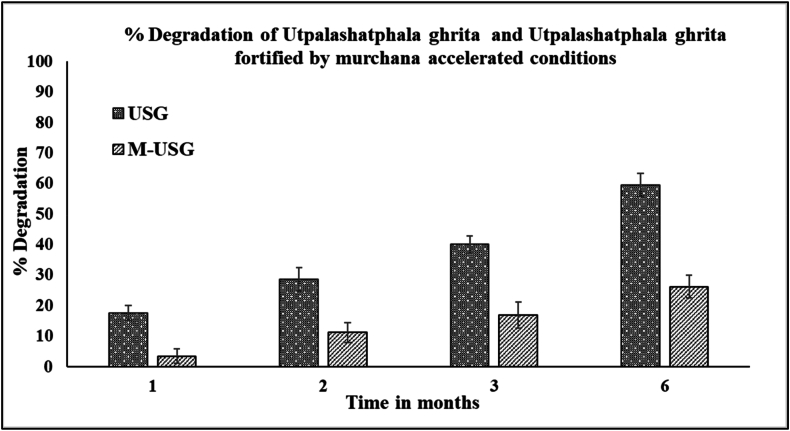
Pictorial representation of stability study of piperine in USG and M-USG at accelerated conditions.

The stability study under accelerated stability conditions showed that USG had a lower PIP content than M-USG. After a month, the PIP content in USG decreased by 17.56 %, whereas in M-USG, it decreased only by 3.48 %. The PIP content decreased by 28.58 % in USG and in M-USG the percentage degradation was only 11.18 % after two months. PIP degradation rose to 40.12 % in USG and 16.90 % in M-USG after three months. After six months, the PIP content dropped by 59.48 % in USG, while in M-USG the reduction in content of piperine was only 26.23 % in M-USG as shown in Fig. 5. The accelerated stability study reveals that M-USG maintains significantly higher PIP content than USG over six months, with notably lower degradation at each time point. By six months, USG lost nearly 60 % of PIP, while M-USG degradation was only 26.23 %. This underscores the superior protective effect of the murchita process in preserving the PIP content. Accelerated stability study employs harsh conditions of temperature and humidity. The results of accelerated stability study thus helps to predict the long term stability and helps in identifying potential degradation products within a shorter time frame. This study helps to validates the long term stability of PIP on storage.

##### 3.2.2.4 Refrigerated condition

**Fig. 6:**
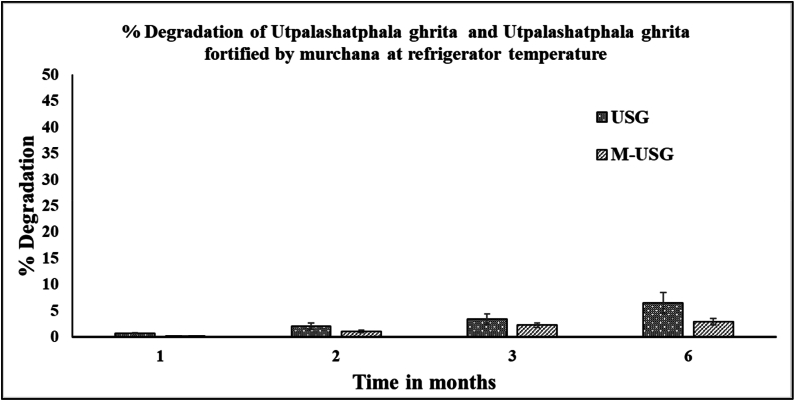
Pictorial representation of stability study of piperine in USG and M-USG at refrigerator condition.

Both USG and M-USG were stable at refrigerated condition. After a month, the % degradation of PIP was only 0.67 % and 0.17 % for USG and M-USG respectively. After two months it was 2.04 and 1.07 % for USG and M-USG respectively. The percentage degradation was almost similar at the 3 month's time point. After six months, the PIP content dropped by 6.48 % in USG while it was only 2.93 % in M-USG, as shown in Fig. 6. The data shows that degradation of PIP is very minimal in both USG and M-USG.

Statistical comparisons of the degradation rates between USG and M-USG under various stress conditions were conducted using ANOVA. The analysis revealed that M-USG demonstrated a significantly lower degradation rate than USG (P < 0.0001), with a mean difference of 22.64 **(**Table S1). This result was consistent across all tested conditions, as indicated by the absence of a significant interaction effect (P = 0.207). Further quantitative analysis of degradation kinetics revealed that M-USG has an extended half-life (12.97 months) compared to USG (4.58 months), indicating a more gradual loss of active components over time (Fig. S1). The greater stability of M-USG strongly supports its potential to maintain the integrity and quality of ghrita more effectively, thereby prolonging its shelf life.

The observed stability of PIP in M-USG could be credited to the murchita process. The murchita process of preparing ghrita involves specific collection of herbs, each containing key active constituents that contribute importantly to the stability and quality of the final formulation. *Haritaki* (*Terminalia chebula*) is rich in hydrolysable tannins such as chebulinic acid, chebulagic acid, chebulic acid, gallic acid, and ellagic acid [20]. These polyphenolic compounds exhibit strong antioxidant activity by scavenging free radicals and chelating metal ions in lipids [21]. This antioxidant property is crucial in protecting ghee's fatty acids from lipid peroxidation, thereby reducing rancidity and prolonging shelf life [22]. It is a well-known fact that piperine is prone to oxidative degradation. Polyphenols prevent this oxidative degradation of piperine by scavenging free radicals. It is also reported that polyphenols can non covalently interact with piperine through ℼ-ℼ stacking between aromatic rings forming protective molecular complexes, which reduces piperine's exposure to degrading agents like water and oxygen [23]. These reports support the stability of PIP in M-USG observed in our study, proving that murchita process helps in protecting PIP from degradation. Classical Ayurvedic texts, including the Siddha Yoga *Sangraha*, mention *Haritaki* as essential in stabilizing ghrita by controlling “*Ama*” (toxins) and preventing spoiling odors. *Vibhitaki* (*Terminalia belerica*), closely related to *Haritaki*, also contains tannins and phenolic compounds with antioxidant properties that synergize to maintain the hue, aroma, and texture of the ghrita. In Charaka Samhita [24]*,* *Vibhitaki* is described as “*Pachana*” (digestive) and “*Shodhana*” (purifying), which in the context of murchita ghrita relates to the removal of impurities and enhancing stability [25]. *Amalaki* (*Emblica officinalis*), a notable source of vitamin C (ascorbic acid) and various polyphenols such as gallic acid and ellagic acid, also provide potent antioxidant activity [26]. These constituents help neutralize free radicals, thus preventing oxidative spoilage in the ghee. Studies on Amalaki extract demonstrated its ability to prevent oxidative rancidity in lipid-based formulations, thus preserving the freshness and nutritional quality of ghrita. Ayurveda texts, including the Bhavaprakasha, highlight Amalaki for its rejuvenating (*Rasayana*) properties and capacity to maintain product integrity [27]. *Haridra* (*Curcuma longa* or turmeric) provides curcuminoids, primarily curcumin, known for their strong anti-inflammatory and antioxidant effects. Curcumin acts as a potent inhibitor of lipid peroxidation by scavenging free radicals and stabilizing cell membranes. Inclusion of *Haridra* during *murchita* process imparts a characteristic yellow hue to the *ghrita* while chemically stabilizing the lipids [28]. Another ingredient of the murchita process, *Musta* (*Cyperus rotundus*) contributes flavonoids, essential oils, and terpenoids that have preservative and detoxifying properties, helping cleanse and reduce the microbial contamination in the *ghrita* and maintain stability of PIP during storage [29]. Lastly, *Matulunga* (*Citrus medica*) is a rich source of volatile essential oils like limonene and citral and citric acid derivatives [30]. These volatile oils can mask undesirable odors, enhancing the sensory appeal of *ghrita*, while the organic acids neutralize alkaline impurities, which may catalyse degradation of PIP. All these herbs—through their polyphenols, tannins, curcuminoids, flavonoids, essential oils, and acids—create a synergistic effect in the murchita process, inhibiting degradation of PIP, providing it good physical and chemical stability, and prolonging shelf life, while also improving the sensory and therapeutic qualities of the *ghrita*.

#### 3.2.3 Greenness of analytical method

**Fig. 7:**
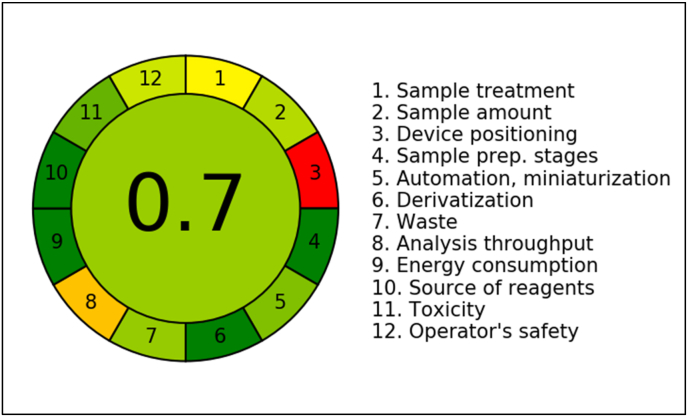
Pictorial representation for greenness profile assessment of LCMS method.

Green analytical chemistry (GAC) focuses on minimising the environmental and health impacts with analytical procedures, particularly those involving hazardous organic solvents, energy consumption, or waste generation. The growing awareness of sustainability in laboratory practices has led to the development of various tools to assess and quantify the greenness of analytical methods. One such tool is the AGREE software, which evaluates an analytical method based on the 12 principles of GAC and generates a numerical score between 0 and 1, where a higher score indicates a greener, more sustainable method. In this study, the greenness of the developed LC-MS method for the quantification of PIP in ghrita was assessed using AGREE. As shown in Fig. 7, the method achieved on overall AGREE score of 0.7, suggesting that the analytical procedure is relatively eco-friendly, with minimal environmental impact and acceptable safety for operators. The detailed greenness evaluation of LCMS method has been shown in Fig. S2 which visually represents individual scores on aspects such as solvent toxicity, energy consumption, waste generation, and operator safety. This detailed evaluation allows for transparent validation of the green credentials claimed and helps identify specific areas for improvement. In our method, the presence of chloroform in the extraction step can be a concern for toxicity. In our extraction trials, we tried extraction of PIP from ghee with available nonpolar solvents like ethyl acetate, acetonitrile, acetone and chloroform and got best recovery from chloroform. However, the overall use of chloroform in the whole method is only o.5 mL for the extraction process. Accordingly, the AGREE score of 0.7 is relatively green, showing its environmental friendliness.

## 4 Conclusion

An LCMS based analytical method was developed and validated for the quantification of PIP from ghee based Ayurvedic preparations such as USG and M-USG. An evaluation of stability of PIP in USG and M-USG under various storage conditions showed that PIP was stable in M-USG, while the PIP content in USG underwent degradation linearly with time. This observation supports the importance of murchita process reported in Ayurvedic literatures in preserving the potency of ghee-based preparations. Among the various storage conditions, the refrigerated condition was found to be the best in protecting the PIP content in the ghrita. At the room temperature the PIP content in the *murchita ghrita* (M-USG) showed only 6.23 % degradation in comparison to the 26.48 % reduction in the PIP content of USG, in which the murchita process was not done. The intermediate and accelerated stability testing also showed a similar trend, with USG showing significantly greater degradation compared to M-USG. It was observed that the degradation of PIP from M-USG at all the conditions were within the acceptable limits of ±15 % proving the importance of this method in preserving the active content as described in the Ayurvedic literatures. The accelerated stability study demonstrated that PIP is stable under the harsh temperature and humidity conditions of this study indicating the longer shelf life at the normal storage at the room temperature for the M-USG. The finding from this study clearly indicates that the *murchita* process can enhance the long-term stability of PIP in ghrita-based formulations. The findings of this work is an eye opener for the ayurvedic pharmaceutical companies, who skip the murchita processing step to save time and cost. As a limitation, the six month stability study may not provide an assessment of the multi-year shelf-life typical of Ayurvedic formulations and the study is restricted to chemical stability without in-depth evaluation of biological efficacy or physicochemical changes. Future research focusing on long-term stability, biological activity retention, and scale-up feasibility will be crucial to fully validate the benefits of the murchita process and facilitate integration into standard Ayurvedic pharmacopoeia with enhanced shelf-life assurance.

**Author contributions**

AG: Conceptualization, Methodology / Study design , Software, Formal analysis, Data curation, Writing – original draft, Writing – review and editing, Project administration.

ARM: Conceptualization, Methodology / Study design , Formal analysis,Writing – review and editing, Project administration.

MS: Methodology / Study design , Formal analysis, Project administration.

JDN: Conceptualization, Investigation, Supervision.

SM: Conceptualization, Investigation, Resources, Writing – review and editing, Supervision.

## Declaration of generating AI in scientific writing

The authors have not used any AI tools for writing this manuscript.

## Funding sources

No funding.

## Conflict of interest

The authors declare that they have no known competing financial interests or personal relationships that could have appeared to influence the work reported in this paper.
