# Supplementary material for: Validation of an LCMS method for stability evaluations of piperine in Murchita utpalashatpalaghrita
Source: J Ayurveda Integr Med. 2026 Feb 4;17(1):101286. doi: 10.1016/j.jaim.2025.101286 (PMC12891786; doi:10.1016/j.jaim.2025.101286)
Supplement: Multimedia component 1 [file mmc1.docx]

| **Table Analyzed** | **Grouped: Two-way ANOVA (two data sets)** |  |  |  |  |
| --- | --- | --- | --- | --- | --- |
|  |  |  |  |  |  |
| Two-way ANOVA | Ordinary |  |  |  |  |
| Alpha | 0.05 |  |  |  |  |
|  |  |  |  |  |  |
| **Source of Variation** | **% of total variation** | **P value** | **P value summary** | **Significant?** |  |
| Interaction | 6.806 | 0.207 | ns | No |  |
| Row Factor | 22.53 | 0.0026 | ** | Yes |  |
| Column Factor | 38.21 | <0.0001 | **** | Yes |  |
|  |  |  |  |  |  |
| **ANOVA table** | **SS** | **DF** | **MS** | **F (DFn, DFd)** | **P value** |
| Interaction | 913 | 4 | 228.2 | F (4, 30) = 1.573 | P=0.2070 |
| Row Factor | 3023 | 4 | 755.7 | F (4, 30) = 5.209 | P=0.0026 |
| Column Factor | 5126 | 1 | 5126 | F (1, 30) = 35.33 | P<0.0001 |
| Residual | 4352 | 30 | 145.1 |  |  |
|  |  |  |  |  |  |
| **Difference between column means** |  |  |  |  |  |
| Mean of USG | 104.1 |  |  |  |  |
| Mean of M-USG | 81.49 |  |  |  |  |
| Difference between means | 22.64 |  |  |  |  |
| SE of difference | 3.809 |  |  |  |  |
| 95% CI of difference | 14.86 to 30.42 |  |  |  |  |
|  |  |  |  |  |  |
| **Data summary** |  |  |  |  |  |
| Number of columns (Column Factor) | 2 |  |  |  |  |
| Number of rows (Row Factor) | 5 |  |  |  |  |
| Number of values | 40 |  |  |  |  |

**Supplementary file**

**Table**

Table S1: Statistical comparisons of the degradation rates between USG and M-USG under various stress conditions.

**Figure**

**
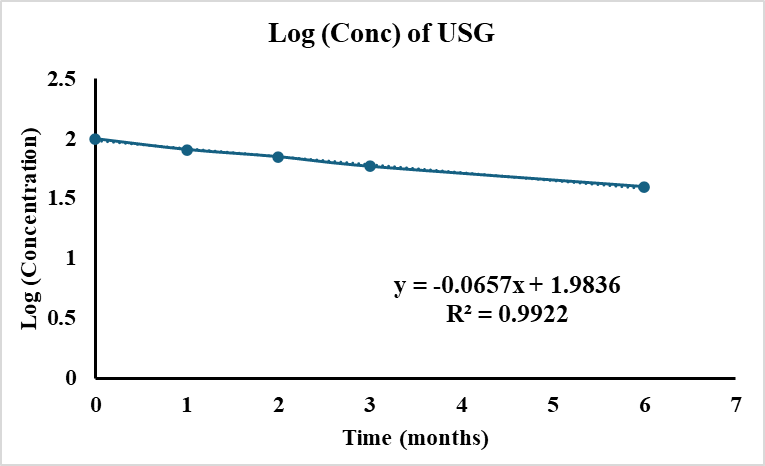
**

**(A)**

**
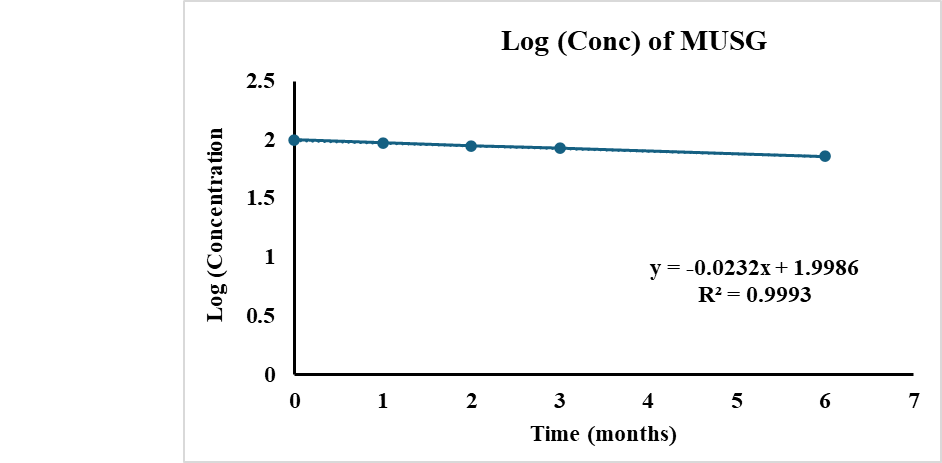
**

Figure S1: Loss of active components over time (A) USG, (B) MUSG.

**(B)**


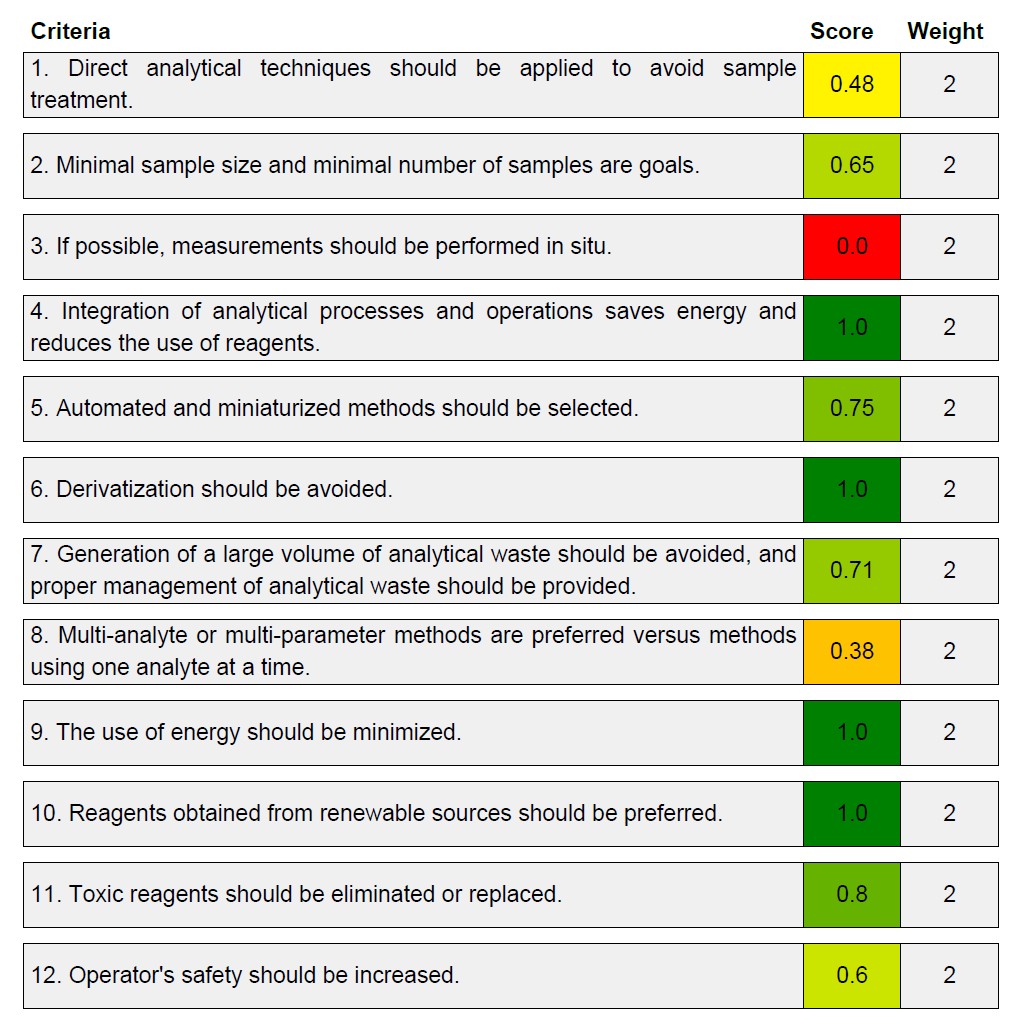
Figure S2: AGREE report of the different factors for the greenness of the LCMS method.
